# Managing Adult Obese Patients at Primary Health Care Centers in Qassim Province, Saudi Arabia

**DOI:** 10.7759/cureus.51704

**Published:** 2024-01-05

**Authors:** Abdulmajeed A Alnughaymishi, Chandra Sekhar

**Affiliations:** 1 Family Medicine, Family Medicine Academy, Qassim Health Cluster, Buraydah, SAU

**Keywords:** saudi arabia, obesity training, practice, attitude, knowledge, phcc physicians

## Abstract

Background

Overweight and obesity have become a global health problem. This study aims to reduce the same problem, primarily with all levels of physicians and the hidden responsibility of all other allied health care workers and communities, including families and individuals.

Objective

The objective of this study is to determine the knowledge, attitude, and practice among the physicians toward the management of adult obesity at primary health care centers (PHCCs) of Qassim Province, Saudi Arabia.

Methods

A cross-sectional study was conducted among the 140 physicians working at the PHCC of Qassim through a self-administered questionnaire. A simple random method was used for the PHCC selection, and all the physicians were included at the PHCC level. Data was collected, cleaned, and analyzed with IBM SPSS Statistics, version 21.0 (IBM Corp., Armonk, NY). Necessary statistical tests were applied.

Results

The mean age and standard deviation (SD) in the study population were 34.29 ± 9.42, and 55.7% were males. The mean knowledge score and SD of correct answers were 4.61 ± 1.31. About four or more questions were answered correctly out of six knowledge questions (80.7%, n = 113). Out of 13 questions, the mean ± SD of correct practice questions was 9.88 ± 2.02. The mean ± SD of attitude questions was 8.06 ± 1.13. About 33 (23.6%) of the study population received obesity training courses.

Conclusions

Based on the study results, good knowledge, practice, and attitude were observed among the PHCC physicians of Qassim. However, a smaller number of physicians received obesity training in the study.

## Introduction

According to the World Health Organization (WHO), obesity is defined as abnormal or excessive fat accumulation that presents a health risk. Worldwide obesity has nearly tripled since 1975. Nowadays, obesity is a global health issue from developed countries to developing countries. It could be due to modern lifestyles, and it is a significant risk factor for multiple comorbidities, such as hyperlipidemia, coronary artery disease, hypertension, osteoarthritis, obstructive sleep apnea, type 2 diabetes, and some cancers [1].

Also, obesity has strong evidence of affecting bad outcomes of male and female fertility and psychiatric illnesses such as depression and modesty [2-4]. There is also an association between obesity and low self-esteem [5]. As a result of the increase in obesity in the community, there are more chances of comorbidity, which results in less productivity, a decrease in life expectancy, and thereby leads to low quality of life.

Regardless of the harm of obesity on health, obesity itself and its comorbidities are responsible for a considerable cost to both the health system and society [6]. As Saudi Arabia has a prevalence of obesity greater than the global prevalence of obesity, perhaps due to high socioeconomic status, we see it as an alarming health issue that needs to be analyzed [7]. One of the measurements in controlling obesity is through primary health care, as it has easy accessibility and well-trained doctors. Primary health care doctors are the cornerstone in controlling, preventing, and treating obesity.

A study conducted in Saudi Arabia concluded that the physicians working in PHCC have an acceptable level of attitude and practice but need more education and training in managing and preventing obesity [8]. Another study revealed that training is needed with respect to managing obesity in Saudi Arabia, and physicians have acceptable knowledge of the management of obesity and overweight. However, they need more training regarding the prevention and management of obesity [9].

Also, a study was conducted on Hungarian general practitioners’ knowledge, attitude, and practice, and the study concluded that there is a need for more education, guidelines, and higher community involvement to improve obesity management [10]. Another study conducted in New York City, USA, concluded that there is evidence of attitudinal differences toward obesity and obese patients between those physicians who self-identify as rural versus non-rural [11]. Obesity management guides would help to increase knowledge and reduce weight-related stigmatization in primary care, thereby improving medical care for obese and overweight patients [12].

To reduce the problem, community participation and health care professionals take responsibility for educating the community and suggesting some remedial measures at the appropriate time and during the first level contact at primary health care centers (PHCCs). Literature suggests that primordial prevention strategies should be adopted at the earliest to prevent the obesity problem in our community and promote health promotional measures. Given the above situations and circumstances, the present study was planned to evaluate the knowledge, attitude, and practice of physicians toward the management of obesity patients at PHCCs.

## Materials and methods

Study setting and target population

Physicians working at the PHCCs of Qassim Province, Saudi Arabia, were selected for the study.

Study design

A cross-sectional study was conducted among primary health care physicians.

Questionnaire and data collection tool

The questionnaire was developed based on the previous questionnaires [10,13], our research idea supported with a literature review, and also from the experts’ opinions of the Research and Innovation Unit at Family Medicine Academy, Qassim. Before the finalization of the study, an email was communicated to the principal investigator in the Hungary study, and later, a new questionnaire was developed. The questionnaire consists of four parts. The first part deals with socio-demographic variables like age, gender, qualification, and position of the participant. The second part denotes physician knowledge questions about obesity, the third part comprises attitude questions, and the fourth part is incorporated as practice questions of physicians about obesity management at PHCCs. 

After the completion of the questionnaire, a pilot test was done on 10 study participants, and the sample was not included in our main study sample. It was distributed personally to the physicians working at PHCCs (self-administered questionnaire). The principal investigator and data collectors are available during the questionnaire filling to avoid confusion or some doubts about the questionnaire. The questionnaire consists of mostly closed-ended questions and some open-ended questions like age. After completion of the self-administered questionnaire by the participant, the principal investigator fills the final score of knowledge, attitude, and practice domain scores of each physician.

The second section deals with knowledge questions, including the definition of obesity, therapy, referral, and indications of surgery and behavioral modification. Similarly, some attitude questions include the following: I consider obesity as a disease, I recommend a weight loss program to my patients, I am comfortable in treating obese patients, I will monitor the BMI and waist circumference of my patients, and I recommend physical activity importance to my patients. Lastly, some practice questions include the history of anthropometry, ready-to-treat obesity patients at PHCC, calculation of BMI and obesity diagnosis, do you use a lab to diagnose obesity, interest in taking counseling sessions with obese patients, and referral to a dietician and specialized clinics. 

In our study, we kept six knowledge questions, nine attitude questions, and 13 practice questions. Participants who answered four and above knowledge questions correctly were labeled as having good knowledge, and participants who answered less than four questions correctly were labeled as having poor knowledge. Also, of the 13 practice questions, participants who answered seven questions correctly were considered good practice, and those who answered less than seven questions correctly were considered poor practice. Similarly, for the nine attitude questions, those who answered five attitude questions correctly were considered as having a good attitude, and those who answered less than five questions correctly were labeled as having a poor attitude. 

Sample size and sampling method

Based on the General Authority for Statistics (2017), 168 PHCCs function in the Qassim province, and close to 550 primary health care physicians work across the province [14]. Close to a 3:2 ratio of male physicians to female physicians are working at various PHCCs. In our study, we plan to take 25% of the total primary health care physicians, which corresponds to 140 physicians. For the selection of PHCC physicians, a simple random method was used from the sample frame. In the Qassim province, nearly 80% of the population is from the cities of Buraidah, Unaizah, and Al Rass. Hence, in our study, we will cover 140 physicians from the PHCCs of the three cities as the time-constrained during the three-year Family Medicine program. For the selection of physicians at the PHCC, all the physicians were physically present on the day the data collectors visited. Before the data collection, a questionnaire was briefed and sensitized to the data collectors.

Inclusion criteria

Physicians were present on the day of collection of our visit. Information was collected about only adult obese patients’ management.

Exclusion criteria 

Physicians working at hospitals and physicians who are working at private clinics were excluded. Physicians who were on vacation and not interested in the study participation were also excluded. 

Ethical considerations

The institutional ethical clearance certificate was obtained from the Qassim Regional Ethics Committee with an approval number of 607-44-2621. The data collection process was started after ethical approval. Before visiting the concerned PHCC, permission was obtained from the regional PHCC director. One day before, information was given to concerned PHCC physicians to ensure their presence for data purposes. Informed consent was obtained from each participant. Confidentiality of the information was maintained. Personal information and the privacy of the participants are protected.

Statistical analysis

Data was entered in the IBM SPSS Statistics, version 21.0 (IBM Corp., Armonk, NY). Percentages and means were calculated for the descriptive variables. The chi-square test was applied for categorical analysis. The statistical significance of the test was considered as a probability (P) value less than or equal to 0.05.

## Results

The self-administered questionnaire was distributed to 180 physicians, and the physicians who responded were 140. The response rate in the study population in our study was 78% (140/180). The mean years of experience and standard deviation (SD) of the study population were 7.48 ± 7.78. The mean knowledge score and SD of correct answers were 4.61 ± 1.31, and out of six knowledge questions, the number of physicians who answered about four or more questions correctly was 113 (80.7%). The mean number of correct practice questions and their SD are 9.88 ± 2.02 (out of 13 questions). Concerning attitude, the mean and SD of correctly answered attitude questions were 8.06 ± 1.13 (out of nine questions). In the study population, about 33 (23.6%) received obesity training courses.

Table 1 depicts that the mean age and SD in the study population were 34.29 ± 9.42, and 61.4% were in the age group of <35 years. Just more than half of the physicians (53.6%) were married. In the study population, about 57.9% were Saudi physicians.

**Table 1:** Demographic characteristics among the physicians in the Qassim Province, Saudi Arabia

Variables	Number of participants	Percentage
Nationality		
Saudi	81	57.9
Non-Saudi	59	42.1
Age ± SD	34.29 ± 9.42	
Age category		
≤35 years	86	61.4
36-45 years	30	21.4
>46 years	24	17.1
Gender		
Male	78	55.7
Female	62	44.3
Qualification		
Board	20	14.3
Master	6	4.3
Diploma	4	2.9
Bachelor	110	78.6
Position of the doctor		
Consultant	4	2.9
Specialist	21	15
General physician	115	82.1
Marital status		
Married	75	53.6
Single	64	45.7
Divorced or widow	1	0.7

Table 2 states that in the study population, WHO’s definition of obesity is only 73.6%, followed by pharmacological therapy for obesity, which is 70%. Regarding the knowledge about behavior modification in obesity-related questions, 95% of physicians answered correctly. Only 58.6% of physicians answered the question, indicating obese people for bariatric surgery.

**Table 2:** PHCC physicians’ knowledge about adult obesity management status in the study population

Knowledge about obesity management	Correct answer	Wrong answer
WHO's definition of obesity	103 (73.6%)	37 (26.4%)
When will you indicate pharmacological therapy for obese patients	98 (70.0%)	42 (30%)
When you refer obese patients to specialized clinics	119 (85.0%)	21 (15%)
When do you indicate the obese person for the bariatric surgery	82 (58.6%)	58 (41.4%)
What do you mean by behavior modification in obesity	133 (95.0%)	7 (5%)
What is the correct category for overweight and obesity based on BMI	116 (82.9%)	24 (17.1%)

Table 3 revealed that the physician’s practice for obese patients to take a low-calorie diet was 97.1%, and physicians’ advice on exercise for obese people was 94.3%. About questions related to prescribing medication for obesity, only 30% of physicians responded. About 62.9% of physicians used a person’s appearance to diagnose obesity.

**Table 3:** PHCC physicians’ practice component question frequencies in the study population

Practice questions about obesity	Yes	No
Are you taking all history about BMI, waist circumference, and medical and non-medical causes of obesity?	99 (70.7%)	41 (29.3%)
Do you treat obese patients at the primary health care center?	90 (64.3%)	50 (35.7%)
Do you consider height and weight sufficient to diagnose obesity?	117 (83.6%)	23 (16.4%)
Do you use appearance to diagnose obesity?	88 (62.9%)	52 (37.1%)
Do you use laboratories to diagnose obesity?	108 (77.1%)	32 (22.9%)
Do you investigate the secondary cause of obesity?	104 (74.3%)	36 (25.7%)
Are you providing counseling sessions about obesity reduction?	119 (85.0%)	21 (15%)
Do you advise your obese patient to exercise?	132 (94.3%)	8 (5.7%)
Do you advise your obese patient to take a low-calorie diet?	136 (97.1%)	4 (2.9%)
Do you talk about obesity to obese patients visiting for other complaints?	127 (90.7%)	13 (9.3%)
Do you prescribe medication for obese patients?	42 (30.0%)	100 (70%)
Do you refer obese patients to a dietitian clinic?	108 (77.1%)	32 (22.9%)
Do you refer obese patients to specialized clinics?	107 (76.4%)	33 (23.6%)

Table 4 depicts that 76.4% of physicians responded to the questions related to the physicians’ attitude toward monitoring BMI and waist circumference for obesity. About 83.6% of physicians responded on how they act as a model for maintaining normal weight questions. Overall, 97.1% of physicians recommended weight loss programs to their obese patients.

**Table 4:** PHCC physicians’ attitude toward obesity parameter frequencies in the study population

Attitude of physician	Yes	No
I consider obesity as a disease	126 (90.0%)	14 (10%)
I will recommend weight loss program to my patients	136 (97.1%)	4 (2.9%)
I feel comfortable in treating my obese patients	120 (85.7%)	20 (14.3%)
I suggest obese patients to seek dietician advice	133 (95.0%)	5 (5%)
I refer obese patients to obesity-specialized clinics	120 (85.7%)	20 (14.3%)
I will monitor BMI and waist circumference readings on every visit	107 (76.4%)	33 (23.6%)
I recommend physical activity to my obese patients	136 (97.1%)	4 (2.9%)
I increase the nutrition awareness of my patients to reduce obesity	134 (95.7%)	6 (4.3%)
Physician acts as a model for maintaining normal weight	117 (83.6%)	23 (16.4%)

Table 5 states that obesity knowledge is further classified based on the number of questions answered by the PHCC physicians. Out of six knowledge questions, physicians who answered four or more questions correctly were labeled as having good knowledge, and those who answered less than four questions correctly were labeled as having poor knowledge. Good knowledge about obesity was observed among male physicians at 82.1% and female physicians at 79% (P > 0.05). Similarly, there was no significant association between the nationality, qualification, and position of the physician in the management of obesity knowledge.

**Table 5:** Demographic factor association with PHCC physicians’ knowledge about obesity management

Sociodemographic factors	Good knowledge	Poor knowledge	X^2^ test	P-value
Male	64 (82.1%)	14 (17.9%)	0.202	0.653
Female	49 (79%)	13 (21%)		
Saudi	68 (84%)	13 (16%)	1.293	0.255
Non-Saudi	45 (76.3%)	14 (23.7%)		
Board	14 (70%)	6 (30%)	3.478	0.324
Master	4 (66.7%)	2 (33.3%)		
Diploma	4 (100%)	0 (0%)		
Bachelor	91 (82.7%)	19 (17.3%)		
Consultant	3 (75%)	1 (25%)	1.512	0.469
Specialist	15 (71.4%)	6 (28.6%)		
GP	95 (82.6%)	20 (17.4%)		

Table 6 reveals that obesity practice is further stratified based on the number of questions answered by the PHCC physicians. In the study, out of 13 practice questions, physicians who answered seven or more questions correctly were labeled as good practice, and those who answered less than seven questions correctly were labeled as poor practice.

**Table 6:** Demographic factor association with PHCC physicians’ practice about obesity management

Sociodemographic factors	Good practice	Poor practice	X^2^ test	P-value
Male	75 (96.2%)	3 (3.8%)	0.494	0.482
Female	58 (93.5%)	4 (6.5%)		
Saudi	76 (93.8%)	5 (6.2%)	0.557	0.456
Non-Saudi	57 (96.6%)	2 (3.4%)		
Board	20 (100%)	0 (0%)	2.010	0.570
Master	6 (100%)	0 (0%)		
Diploma	4 (100%)	0 (0%)		
Bachelor	103 (93.6%)	7 (6.4%)		
Consultant	4 (100%)	0 (0%)	1.602	0.449
Specialist	21 (100%)	0 (0%)		
GP	108 (93.9%)	7(6.1%)		

Table 6 revealed that board, master, and diploma qualification physicians had 100% (seven out of 13 questions) obesity practice compared to bachelor degree physicians (93.6%). Similarly, the position of physicians, consultants, and specialist physicians had 100% practice (seven out of 13 questions) in obesity management compared to general physicians (93.9%).

Table 7 depicts that the obesity management attitude of physicians is further divided based on the number of questions answered by the PHCC physicians. In the current study, out of nine attitude questions, physicians who answered five or more questions correctly were labeled as having a good attitude, and those who answered less than five questions correctly were labeled as having a poor attitude. There was no significant difference in the obesity management attitude of physicians with gender, nationality, qualification, and position (P > 0.05).

**Table 7:** Demographic factor associated with PHCC physicians’ attitudes about obesity management

Sociodemographic factors	Good attitude	Poor attitude	X^2^ test	P-value
Male	77 (98.7%)	1 (1.3%)	0.027	0.870
Female	61 (98.4%)	1 (1.6%)		
Saudi	80 (98.8%)	1 (1.2%)	0.051	0.821
Non-Saudi	58 (98.3%)	1 (1.7%)		
Board	20 (100%)	0 (0%)	0.553	0.907
Master	6 (100%)	0 (0%)		
Diploma	4 (100%)	0 (0%)		
Bachelor	108 (98.2%)	2 (1.8%)		
Consultant	4 (100%)	0 (0%)	0.441	0.802
Specialist	21 (100%)	0 (0%)		
GP	113 (98.3%)	2 (1.7%)		

## Discussion

The current study was conducted among primary health care physicians about knowledge, practice, and attitude toward the management of obesity at their respective centers from September 2022 to November 2023. People have an affinity for adopting good health promotional measures through the physician’s advice. Also, the population expects the ideal body weight from physicians and their timely advice about obesity and its consequences. The mean age and SD in the current study population were 34.29 ± 9.42. A study conducted in the eastern province stated that the mean age and SD in their study were 40.77 ± 8.26. The mean age in our study could be lower due to age shift and the early age to start a career due to Saudization, as per the Vision 2030 initiative in Saudi Arabia [8].

In our current study, nearly 61% of PHCC physicians were in the age group of 25-35 years, whereas the study conducted in Riyadh, Saudi Arabia, mentioned that 47% of physicians were in the age group of 25-35 years. The variation in the percentage could be due to a study that was conducted way back in 2014 (nine years ago), and presently, many young graduates are joining PHCC and are also inclined to pursue higher studies [13]. 

An interesting finding in the current study population is that only 33 out of 140 (23.6%) physicians received obesity training courses. A study conducted in Saudi Arabia found relatively less prevalence of training about obesity management among the PHCC physicians [9] and also among the PHCC physicians of Kuwait [15]. Another study conducted in Saudi Arabia by Alomary et al., which was published in 2016, stated that 15% of male physicians and 16% of female physicians were trained in the management of obesity [13]. Lack of training and periodic training about obesity leads physicians to lose confidence in obesity management, have low motivation, and not treat it with interest.

About 95% of physicians answered correctly with knowledge about behavior modification in obesity questions. Some studies stated that long-term behavior modification plays a vital role in the management of obese patients [8,16]. A study conducted in Riyadh stated that physician knowledge and lack of training are major barriers to nutritional counseling and practice. On the whole, regular training, updates, and implementation play a role in the reduction of obesity cases at PHCCs, indirectly at the community level [17].

Out of six knowledge questions, the mean knowledge score and SD of correct answers were 4.61 ± 1.31 (n = 113, 80.7%). Physicians who answered four questions correctly will be considered to have good knowledge. On the whole, overall knowledge about obesity management is good, but individual questions, like indications for surgery for obese patients and pharmacological therapy for obese patients, need improvement. Good knowledge about obesity management was noticed in a study conducted in Saudi Arabia by primary health care physicians [13] and also in other international studies conducted in different regions of the Middle Eastern countries like Kuwait and various parts of the world, including France [15,18-20].

Out of 13 practice questions, the mean number of correct practice questions and their SD are 9.88 ± 2.02 observed in the present study. A study conducted in Germany among primary care physicians stated that a more positive attitude of stakeholders of health care professionals provides a better platform for obesity management among adults [12,21]. In relation to practice questions about the diagnosis of obesity, about 83.6% (n = 117) of physicians gave correct practice. To substantiate the present finding, a study conducted in Al Khobar, Saudi Arabia, stated that about 83.1% (4.44 ± 0.88) of physicians correctly considered a diagnosis for obesity [8].

In relation to nine attitude questions, the mean and SD of attitude correct answer questions are 8.06 ± 1.13 in the current study. Of the attitude questions, one of the important questions is lifestyle modification for the obesity question, for which about 136 (97.1%) of PHCC physicians gave the correct attitude in the current study. A study conducted in the Aseer region, Saudi Arabia, mentioned that 11% of physicians gave a negative response about their attitude toward the management of obesity [22].

Regarding physical activity advice to patients, more than three-fourths of physicians (76.9%) had the correct attitude toward obesity prevention, and this was observed in the year 2014. By this time, the percentage would have increased and lifestyle modification has a great impact on the reduction of obesity [8,23]. This fact was observed in many studies conducted in western England [16], France [18], Israel [24], and Australia [25]. All these studies highlighted that preventive strategies are better than therapeutic approaches in preventing obesity. Also, as lifestyle therapy suggests, behavioral modification is useful in the management of obese patients [16,18,24,25].

Some of the limitations observed in the study were that covering three cities of PHCC consumed more time, and some doctors were not cooperative in filling out the questionnaire despite requests. As our study is a self-administered questionnaire, there is a possibility of misunderstanding some questions by the participants, and to overcome this issue, the principal investigator and data collectors are available during the filling of the questionnaire.

## Conclusions

Based on the study findings, close to one-fourth of the physicians received training on obesity management. In the obesity knowledge domain, four-fifths of the physicians have good knowledge, and close to the same percentage was observed in the practice domain. Regarding attitude toward monitoring BMI and waist circumference readings on every visit, only three-fourths of the physicians responded. As our sample is small, the generalizability of study findings requires more similar studies. More training on obesity management is required for PHCC physicians.
